# Jampersal implementation in primary care, case studies on integrated delivery system of antenatal care in puskesmas and midwives in independent practice in Kecamatan Moyudan, Kabupaten Sleman

**DOI:** 10.1186/1471-2458-14-S1-O4

**Published:** 2014-01-29

**Authors:** Veronika Evita Setianingrum, Mubasyisyr Hasanbasri, Mohammad Hakimi

**Affiliations:** 1 Dinas kesehatan Sleman, Yogyakarta 55511, Indonesia; 2 Faculty of Medicine, Universitas Gadjah Mada, Yogyakarta 55281, Indonesia

## Background

The Indonesian government launched Jampersal programme or labour insurance in early 2011 as one of the efforts to reduce maternal and infant mortality in Indonesia, which aimed at providing financial guarantees for expectant mother to receive antenatal, delivery and postnatal care from health workers in health facilities. The tendency of developing countries in its partial health care system and the large degree of market mechanism, caused imbalance in the quality and ability to access health services among the poor and the rich. Differences in the rules imposed by the Department of Health Sleman in antenatal care services at puskesmas and independent practice midwives led to differences in both the quality of services and costs. This study examined puskesmas which makes efforts on reforming/innovating the health care system based on patient needs. Puskesmas Moyudan performs integration in antenatal care services for Jampersal participants, to ensure expectant mother receive quality and comprehensive antenatal care.

## Materials and methods

This was a descriptive study with qualitative methods, using case study design. In-depth interviews were conducted in 15 respondents, consisting of Basic Medical Section Chief of Sleman Health Department, Jampersal Verifier team of Sleman Health Department, 1 puskesmas midwife coordinator, 4 independent practice midwives, expectant mothers and 8 Jampersal participants. Observation and document search were conducted to complement the data. Integration of antenatal care services was assessed from service quality, cost reduction and the satisfaction of the expectant mothers of Jampersal participants.

## Results

Integration of antenatal care in puskesmas Moyudan included two types of integration, the integration of services between puskesmas and midwives in independent practice, and the integration of antenatal care services in packages. In this package system, Jampersal participants received antenatal care package in the form of obstetric and physical examination, dental consultations, nutritional counselling, psychological counselling and laboratory tests as well as mother’s class. In terms of accessing this service pack, expectant mothers were not charged. The expectant mothers of Jampersal participants were generally satisfied with this integrated antenatal care services, but still the speed of service and officers assurance are yet in line with the expectations.

## Conclusions

Although antenatal care services integration between puskesmas and midwives in independent practice is informal, but with the implementation of the strategy, the improvement of the quality of antenatal care services and cost efficiency can be achieved.

